# Using Deep Learning to Identify Utility Poles with Crossarms and Estimate Their Locations from Google Street View Images

**DOI:** 10.3390/s18082484

**Published:** 2018-08-01

**Authors:** Weixing Zhang, Chandi Witharana, Weidong Li, Chuanrong Zhang, Xiaojiang Li, Jason Parent

**Affiliations:** 1 Department of Geography, University of Connecticut, Storrs, CT 06269, USA; weixing.zhang@uconn.edu (W.Z.); weidong.li@uconn.edu (W.L.); 2 Center for Environmental Science and Engineering, University of Connecticut, Storrs, CT 06269, USA; 3 Connecticut State Data Center, University of Connecticut, Storrs, CT 06269, USA; 4 Department of Natural Resources and the Environment, University of Connecticut, Storrs, CT 06269, USA; chandi.witharana@uconn.edu (C.W.); jason.parent@uconn.edu (J.P.); 5 Eversource Energy Center, University of Connecticut, Storrs, CT 06269, USA; 6 MIT Senseable City Lab, Massachusetts Institute of Technology, Cambridge, MA 02139, USA; xiaojian@mit.edu

**Keywords:** deep learning, utility pole, infrastructure mapping, Google Street View, line-of-bearing measurement, object detection

## Abstract

Traditional methods of detecting and mapping utility poles are inefficient and costly because of the demand for visual interpretation with quality data sources or intense field inspection. The advent of deep learning for object detection provides an opportunity for detecting utility poles from side-view optical images. In this study, we proposed using a deep learning-based method for automatically mapping roadside utility poles with crossarms (UPCs) from Google Street View (GSV) images. The method combines the state-of-the-art DL object detection algorithm (i.e., the RetinaNet object detection algorithm) and a modified brute-force-based line-of-bearing (LOB, a LOB stands for the ray towards the location of the target [UPC at here] from the original location of the sensor [GSV mobile platform]) measurement method to estimate the locations of detected roadside UPCs from GSV. Experimental results indicate that: (1) both the average precision (AP) and the overall accuracy (OA) are around 0.78 when the intersection-over-union (IoU) threshold is greater than 0.3, based on the testing of 500 GSV images with a total number of 937 objects; and (2) around 2.6%, 47%, and 79% of estimated locations of utility poles are within 1 m, 5 m, and 10 m buffer zones, respectively, around the referenced locations of utility poles. In general, this study indicates that even in a complex background, most utility poles can be detected with the use of DL, and the LOB measurement method can estimate the locations of most UPCs.

## 1. Introduction

Maintaining the electric grid is a challenging task and accurate maps of utility infrastructure are important for planning and operations, managing risk, and rapidly assessing damages after a storm [1]. However, the lack of exact locations of electric facilities is not uncommon [2]. For example, after hurricane Maria struck Puerto Rico in September of 2017, the lack of accurate maps for buildings, bridges, and electric facilities was considered as a main factor slowing recovery efforts [3]. Mapping utility poles is labor- and time-intense because the process is usually conducted using human interpretation of high spatial-resolution aerial imagery, ground-based field surveys, or unmanned aerial vehicles (UAVs)/helicopters [2,4]. The high degree of labor requirement makes mapping utility poles over large areas a daunting task.

Remote sensing (RS) provides promising solutions for automated detection and mapping of electric facilities. Utility mapping has been explored using optical sensors, on both satellite and aerial platforms [5,6,7,8,9,10,11,12]; synthetic aperture radars (SAR) [13,14], and light detection and ranging (LiDAR) [15,16,17,18,19]. Cetin and Bikdash [4] mapped utility poles using shadow information derived from aerial images and Sun et al. [20] mapped power poles using stereo images. Wang et al. [19] developed a semi-automated method to classify power lines from LiDAR data in urban areas with both precision and recall up to 98%. However, due to the small size of utility distribution poles, the spatial resolution of most satellite platforms is not sufficient for reliable detection [4,21]. The ultra-high spatial resolutions provided by UAV platforms have made them an emerging tool for surveying electric utilities [22,23,24].

Aerial optical imagery can reliably detect utility poles when the spatial resolution is about 30 cm or better. However, complex backgrounds, and varying sunlight intensities and other factors can prevent utility pole detection [21]. In addition, tree cover in forested areas, can prevent detection of utility poles from aerial images [25]. Mobile mapping systems (MPS) can obtain a better view of utility poles that are obscured by tree cover in aerial images [21]. Studies conducted by Cabo et al. [26] and Lehtomäki et al. [27] reported that most vertical pole-like objects are accurately detected from side views acquired from vehicle-based LiDAR. Cheng et al. [28] developed a voxel-based hierarchical method to extract power lines from vehicle borne LiDAR data in urban area and reported that the correctness and completeness of the exacted power line points were 99% and 93%. Guan et al. [29] mapped power lines and towers using a step-wise method (including off-road point extraction, power-transmission line extraction, and power-transmission line fitting) from vehicle borne LiDAR data with average completeness, correctness, and quality of 92%, 99%, and 91%. The disadvantages of MPS include their high costs of data collection and the massive sizes of their point cloud, which can be challenging to process efficiently [30].

Google Street View (GSV) is an open image collection of panoramic views with estimated accurate geolocation information using GPS, wheel encoder and inertial navigation sensor along streets acquired on mobile platforms (including car, trekker, tricycle, walking, and boat, etc.) [31], which have been increasingly used to enrich geographic information, such as urban greenery [32,33], land use classification [34,35], and shade provision of trees [36]. Some studies have been carried out using side-view imagery for electric utilities detection and survey. For example, Cheng and Song [37] applied a graph cut segmentation method with a set of prior rules to improve recognition of utility poles. Murthy et al. [38] detected utility poles using a template design from video surveillance on car. Barranco-Gutiérrez et al. [39] presented a method to detect utility poles from complex environment based on color, shape and photometric stereovision using dual cameras. Song and Li [40] developed a sequential local-to-global algorithm to detect power lines from optical images and tested on 160 pictures taken from the ground with 91.95% and 91.33% true positive rates for detecting straight lines and curved lines respectively. However, in general, it is difficult to distinguish utility poles from all vertical pole-like objects along roads even by human interpretation because of their small cross-sectional area [41]. In Sharma et al. [30], a five-stage detection algorithm (including segmentation, block-oriented quadrilateral extraction, quadrilateral shape determination, orientation-based spatial clustering of near-trapeziums, and context-based detection) was developed to detect utility poles in pure side-view images; 70% of poles from 212 frames ground truth were detected. However, the previously used methods are complicated because they involved utilizing a variety of models and algorithms, such as feature segmentation, extractions, filters, detection, and template match, among others.

Deep learning (DL) has shown its powerful ability in computer vision, natural language processing, and many other fields [42,43,44,45,46,47]. However, there are very few published studies on using DL to map or inspect power line components. In Nordeng et al. [48], a faster regional convolutional neural network (R-CNN) was used to detect dead-end body component from high voltage power lines with both detection accuracies and precisions up to 97%. Recently, Nguyen et al. [2] conducted a comprehensive review of automatic power line inspection from the prospective of computer vision and the limitations of current vision-based inspection systems, and they suggested that the DL vision-based unmanned aerial vehicle inspection might be a promising new solution. The objective of this study is to use DL on GSV imagery to map utility poles. We focused on the detection of utility poles with crossarms (UPCs) along roadsides. We used a DL-based object detection algorithm (the RetinaNet object detector [49]) to detect UPCs from GSV images and estimated the locations of detected utility poles using a modified brute-force-based line-of-bearing (LOB) measurement method. Therefore, the primary objective of this study is to explore the use of DL in conjunction with GSV for mapping roadside UPCs.

## 2. Data

### 2.1. Study Area and GIS Data

**Figure 1:**
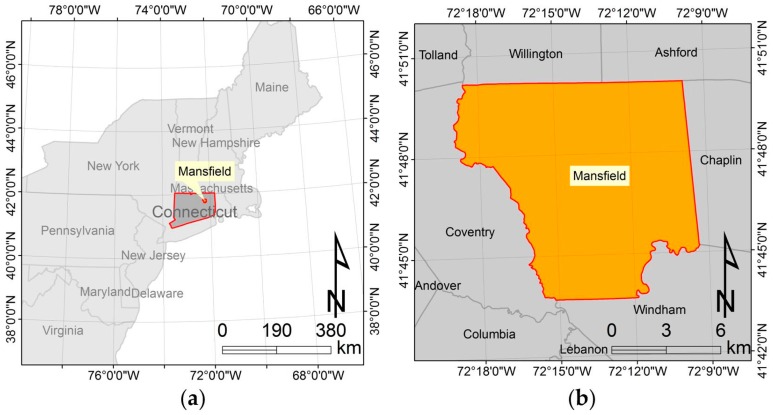
(**a**) Location of the study area in the Northeastern United States; (**b**) location of the study area in the state of Connecticut.

The town of Mansfield (CT, USA) was selected as the study area (Figure 1). Mansfield is a town in Tolland County, located in the Eastern Connecticut, with an area of 118.3 square kilometres based on the 2017 U.S. Census Bureau Topologically Integrated Geographic Encoding and Referencing products. In 2010, Mansfield’s population was 26,543 according to the 2010 U.S. decennial census.

The town boundary and road GIS datasets in vector file format were downloaded from the United State Census Bureau (https://www.census.gov/cgi-bin/geo/shapefiles/index.php). Roads within Mansfield town were extracted and then projected into the NAD83/UTM zone 18N (EPSG: 26918). The extracted road GIS dataset was further pre-processed in order to eliminate duplicated road segments through the following two steps: The first step was to merge all adjacent/overlapped road segments using the “dissolve” tool in ArcGIS software. In the second step, the “Multipart To Singlepart” tool in ArcGIS software was applied to dissolve road segments to generate viewpoints along roads for downloading corresponding GSV via Google API (see Section 2.2 Google Street View imagery). In this study, only major roads in the town were chosen (total length: 91.6 km). A total of 9290 viewpoints were created along the selected roads, at an interval of 10 m, using a geospatial tool called “Create Points on Lines” developed by Ian Broad (http://ianbroad.com/arcgis-toolbox-create-points-polylines-arcpy/). It is worth noting that there are differences between the generated viewpoints and the actual GSV viewpoints in terms of latitude and longitude values because the GSV vehicle does not follow the exactly same routes as the roads in the GIS dataset. A pre-processing solution was used to compromise this mismatching issue (see Section 2.2 Google Street View imagery). Reference UPCs (i.e., the ground truth data) for the whole town were manually mapped using 7.5 cm aerial imagery and checked using GSV. These validation data included 1039 poles, which were located within a 20 m buffer zone around selected roads.

### 2.2. Google Street View Imagery

**Figure 2:**
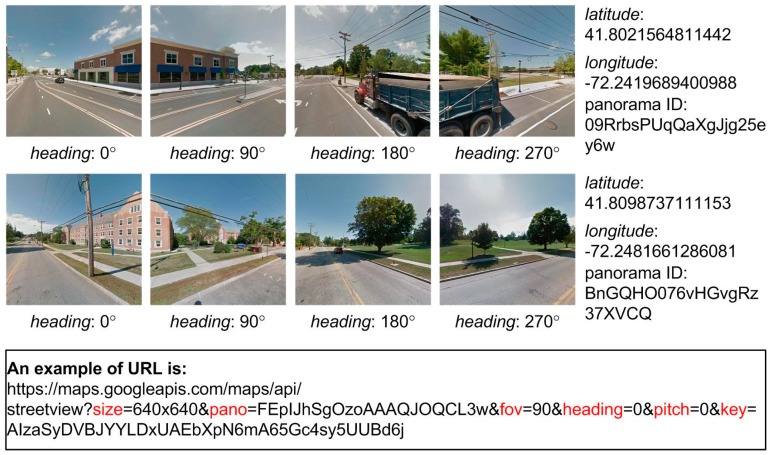
Two sets of GSV images, four per set, were obtained for a view point panorama ID (09RrbsPUqQaXgJjg25ey6w) and a viewpoint point panorama ID (BnGQHO076vHGvgRz37XVCQ) and an example of URL with the parameters highlighted in red color.

**Figure 3:**
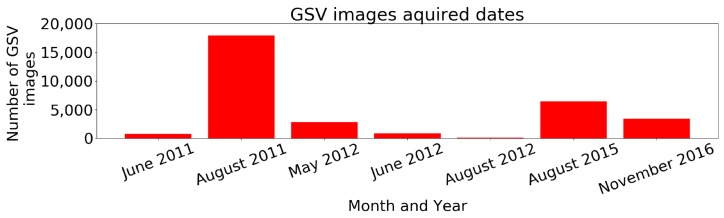
Distribution of GSV images acquisition dates, between 2011 and 2016.

GSV images can detect the orientation of detected UPCs in 360° GSV, similar as the bearing-only sensor, which is commonly used to measure the direction toward features from a robot. Static street view images were downloaded through the GSV image application programming interface (API) by providing uniform resource locators (URL) that embed the appropriate parameter information [50]. The GSV API automatically snaps the requested coordinates to the nearest available GSV viewpoint [50]. In order to acquire accurate latitude and longitude values for each viewpoint of GSV, the “streetview” Python package (https://github.com/robolyst/streetview) was used to convert the requested coordinates into the nearest available GSV coordinates via a list of panorama IDs (i.e., unique identification for each GSV panorama view with acquired date [year, month], latitude, and longitude). The most recent panorama ID was then used as location parameter input. Other required parameters for the URL include size (output size of GSV image), heading (cardinal direction in the range of 360°), fov (horizontal field-of-view angle), pitch (the up or down angle), and API key [50]. Four GSV images were obtained for each view point with a fov of 90° and headings of 0°, 90°, 180°, and 270°, respectively (Figure 2). We developed a Python script to automatically create the URLs and download the 37,160 GSV images needed to cover the study area. The GSV images were acquired between 2011 and 2016 (Figure 3); this range is due to the varying frequencies with which Google updates street view imagery. We cropped the downloaded images to eliminate the Google logos.

### 2.3. Annotation Data

**Figure 4:**
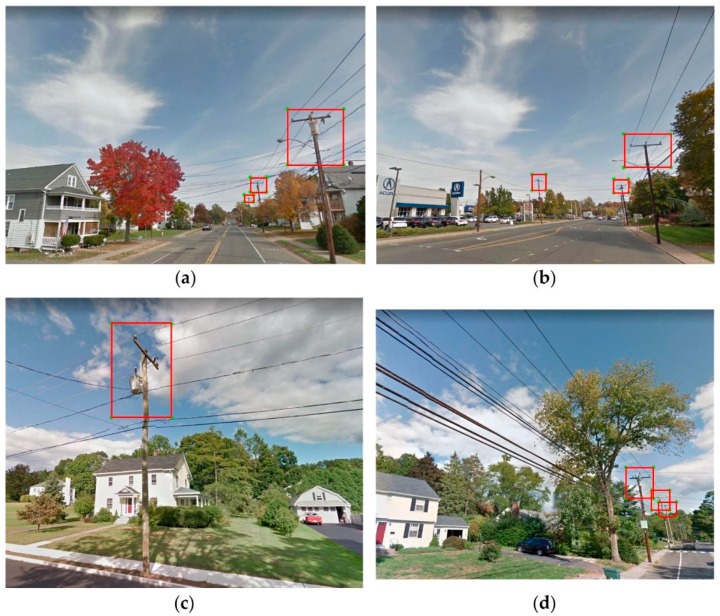
Bounding boxes (red boxes) of labelled utility poles in four GSV images (**a**–**d**).

A large amount of ground-truth data is essential for the deep supervised learning algorithms to be effective [51]. We created 3500 ground-truth data points by manually labelling UPCs in GSV images that were acquired by taking screen captures in Google Maps (Figure 4). In order to enhance the transferability of the method, each training/validation/test image was taken at a random utility pole location in the states of Connecticut, Massachusetts, Maine, New Hampshire, New York, and Texas. The “LabelImg” software (https://github.com/tzutalin/labelImg) was used to annotate the ground-truth images and produce outputs in the format of XML files. This format was used in the Pattern Analysis, Statistical Modelling and Computational Learning project (i.e., PASCAL VOC [http://host.robots.ox.ac.uk/pascal/VOC/]) and the ImageNet database (a large image database for visual object recognition research [http://www.image-net.org]). In general, a full dataset for DL includes a training dataset to “teach” the DL algorithm, a validation dataset for minimizing overfitting of the training data, and a test dataset for assessing the performance. Overall, 2500 annotated GSV images were used as training data, 500 images were used as validation data, and the remaining 500 images were used for accuracy assessment.

## 3. Methodology

### 3.1. General Procedure

**Figure 5:**
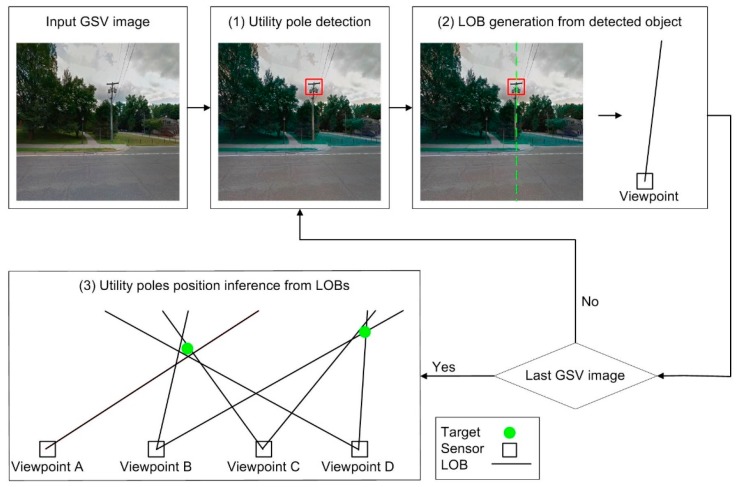
The workflow of mapping utility poles from GSV with deep learning.

Our proposed DL-based automatic mapping method for UPCs from GSV included three main steps (see Figure 5): The first step is to detect UPCs in the GSV images using a trained DL network. The second step is to calculate the azimuth from each viewpoint to the detected UPCs based on the known azimuth angles of the GSV images, relative to their view point locations, and the horizontal positions of the target in the images (Figure 5(2)) using the mean value of two X values of the bounding box. For example, suppose a detected UPC has a bounding box that is centered on column 358 in a GSV image that is centered at 0° azimuth relative to the image viewpoint. Each GSV image contains 640 columns and spans a 90° horizontal field-of-view; thus, each pixel spans 0.14°. The center of the UPC is 38 pixels to the right of the image center (at column 320) and so has an azimuth of 5.3° relative to the image viewpoint. The final step is to estimate the target locations based on the azimuths calculated from the second step (Figure 5(3)).

### 3.2. Deep Learning Algorithm

In this study, the RetinaNet object detector, as described in Focal Loss for Dense Object Detection [49], was used to detect utility poles because of its excellent performance from the prospective of accuracy and computational efficiency [49]. Compared to state-of-the-art two-stage detectors, such as faster R-CNN [52] and Mask R-CNN [53], the RetinaNet object detector is a recently proposed one-stage detector with simpler structure and faster speed, and it can achieve a better accuracy than two-stage detectors. The RetinaNet object detector is based on the one-stage RetinaNet network architecture, which was built on top of the feedforward residual learning network (ResNet) architecture with a Feature Pyramid Network (FPN) [45,49,54]. In terms of structure, a RetinaNet object detector consists of five main components: (1) ResNet as feedforward architecture; (2) FPN as backbone for producing convolutional feature pyramid; (3) region proposal networks (RPN) for generating proposal; (4) fully convolutional network (FCN) as classification subnetwork; and (5) FCN as box regression subnetwork. It is worth noting that the ResNet used in the RetinaNet network was pre-trained on ImageNet (i.e., the transfer learning). For this study, we detected UPCs in GSV images using a Keras (a high-level neural networks API) implementation of a RetinaNet object detector developed by the development team of keras-retinanet on Github (https://github.com/fizyr/keras-retinanet).

### 3.3. Utility Poles Position Inference

Some efforts have been spent in localizing street-level objects using multiple street-view pictures, such as manhole covers, traffic signs. For example, Timofte and Gool [55] developed pipeline to detect and localize manhole covers by using 4 pairs of downward looking stereo cameras and conducting two-steps localization (rough detection from single-view picture and accurate three dimensional (3D) localization from multi-view pictures). Soheilian et al. [56] detected and reconstructed 3D traffic signs and achieved average position accuracy of 3.5 cm by intersecting the corresponding rays from multi-view images and clustering traffic signs candidates. Hebbalaguppe et al. [57] proposed an automated updating system for telecom inventory using object detection and triangulation-based method (stereo-vision distance estimation with the SIFT feature matching algorithm) with GSV. Very recently, it was noticed that Krylov et al. [58] applied a CNN-based sematic segmentation model with a proposed geotagging method to estimate geographic objects’ locations from GSV with two classes—traffic lights and telegraph poles. In this study, we used the RetinaNet object detector and a modified brute-force-based line-of-bearing (LOB) measurement method to localize the position of UPCs from multiple-view GSV images. However, the specific methods we used are different from those used in Krylov et al. [58].

#### 3.3.1. LOB Measurement

**Figure 6:**
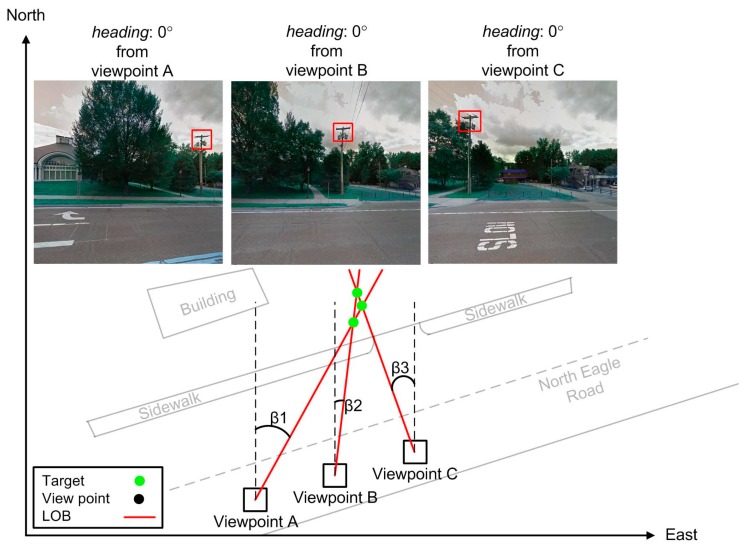
An example of using the bearing measurement to estimate a target location from a sensor from three locations.

The outputs of UPCs detection in GSV images using DL are bounding boxes of detected UPCs, which result from implementation of odometry from monocular vision of GSV images as shown in Figure 6. Therefore, estimating locations of UPCs in pure GSV images is a multiple-source localization problem from passive angle measurements, which has been widely investigated [59,60]. The LOB-based approach is one of three main multiple-source localization approaches [61]. An LOB measurement was applied to estimate the location of a target (i.e., UPC) because detected UPCs are not signal sources such as propagating signal sources whose signal strength can be measured (Figure 6). An LOB measurement does not require as many strict conditions as other methods (e.g., synchronization and power transit) do.

**Figure 7:**
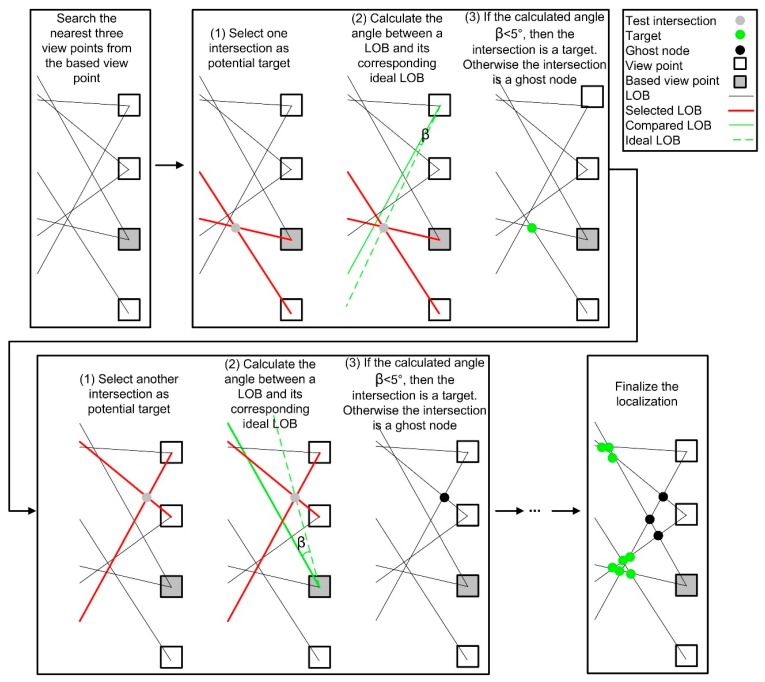
An example of using the brute-force-based three-station cross location algorithm to eliminate ghost nodes from four views with an angle threshold of 5°.

In LOB localization, azimuths from viewpoints of multiple images to a given UPC allow the UPC location to be triangulated (see Figure 6). Ideally, intersection of multiple LOBs is the exact location of the target because the LOBs pass through the target (see Figure 6). However, numerous ghost nodes (i.e., false targets) occur when the LOB measurement is used in a dense emitter environment, as shown in our study for estimating locations of UPCs in GSV images [62] (see Figure 7). As a result, a modified brute-force-based three-station cross location algorithm was utilized to minimize the ghost node problem of multiple-source localization using LOB measurement (Figure 4: source localization from viewpoints A, B, and C), based on two assumptions that targets and sensors are on the xy plane, and all LOB measurements have the same precision [63]. More specifically, the LOB measurement method uses the following steps (Figure 7): (1) for a given viewpoint, find the closest neighboring viewpoints; we tested the performance of the algorithm using 2 to 8 of the closest neighboring viewpoints (i.e., the corresponding number of views is 3 to 9); (2) measure the angles between each pair of LOBs from all viewpoints [64]; (3) check if there are positive associations among LOBs (set as 50 m length) from current viewpoint and its nearest viewpoints [63]; (4) repeat the process from step (1) to step (3) for every intersection point. To be more specific, three positive detections from any three views within an angle threshold (β) produce a positive association among LOBs [63]. Therefore, theoretically, given that detection rates are constant, the number of estimated UPCs increases as the number of views increases based on the probability of combination. For example, assuming the number of all possibilities of UPC estimation is t(t∈N) and the detection rate is constant; then the probability of positive association with 7 views (i.e., $\frac{C\left(7,\;3\right)}{t}$) is greater than the probability of positive association with 4 views (i.e., $\frac{C\left(4,\;3\right)}{t}$).

In this study, nearest viewpoints were selected in order to conduct cross validation. A list of the closest neighboring viewpoints (2, 3, 4, 5, 6, 7, and 8 viewpoints; that is, 3, 4, 5, 6, 7, 8, and 9 views after including the view from the current viewpoint) and angle thresholds (1°, 2°, and 3°) were used for testing to decide if there is a positive association and which threshold performs the best. No more than 9 views were selected for testing mainly because of the length of LOB and the interval of GSV acquisition (10 m). The extreme scenario of 9 views is that 8 viewpoints are on a line and located on one side of the current viewpoint. 80 m is almost the maximum distance requirement for the intersection of two 50-m LOBs. A list of distances (3 m, 4 m, and 5 m) were applied to eliminate ghost nodes that are located too close to the center line of a road. These thresholds allow more relaxation of position inference rule in the LOB measurement. In the process of UPCs detection in GSV, almost parallel rays from camera to long-distance objects can intersect many other rays. Therefore, any object with a width less than 30 pixels was excluded in order to reduce the computation workload of eliminating ghost nodes because the used position inference method is an iterative method.

#### 3.3.2. Multiple LOB Intersection Points Aggregation

**Figure 8:**
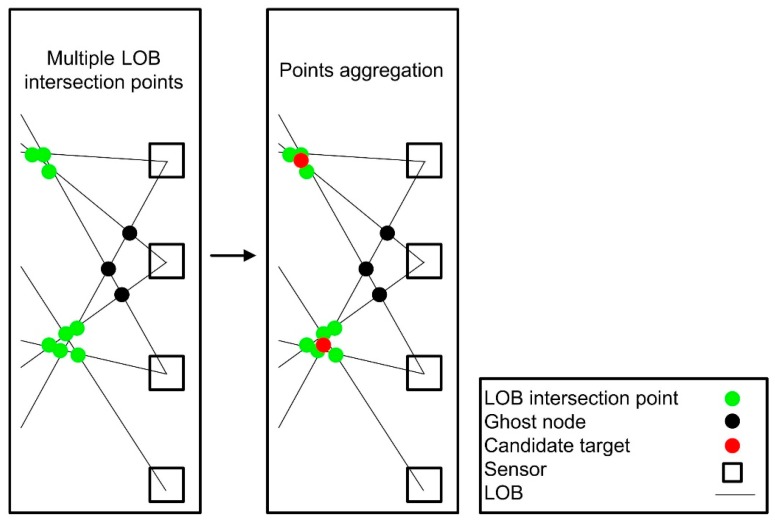
An example of aggregating multiple LOB intersection points.

LOB measurements with a modified brute-force-based three-station cross location algorithm produce multiple LOB intersection points as potential candidates for each utility pole. In order to estimate the most likely location of a utility pole, a geospatial aggregation algorithm with an aggregation distance of 10 m was used to estimate the centroid of clusters of LOB intersection points (see Figure 8). The geospatial aggregation algorithm consists of three main steps: (1) calculate Euclidean distance matrix of all LOB intersection points; (2) cluster LOB intersection points based on the Euclidean distances between LOB intersection points; and (3) calculate the centroid of each cluster of intersection points (Figure 8).

## 4. Experiments and Results

### 4.1. Experiments

**Figure 9:**
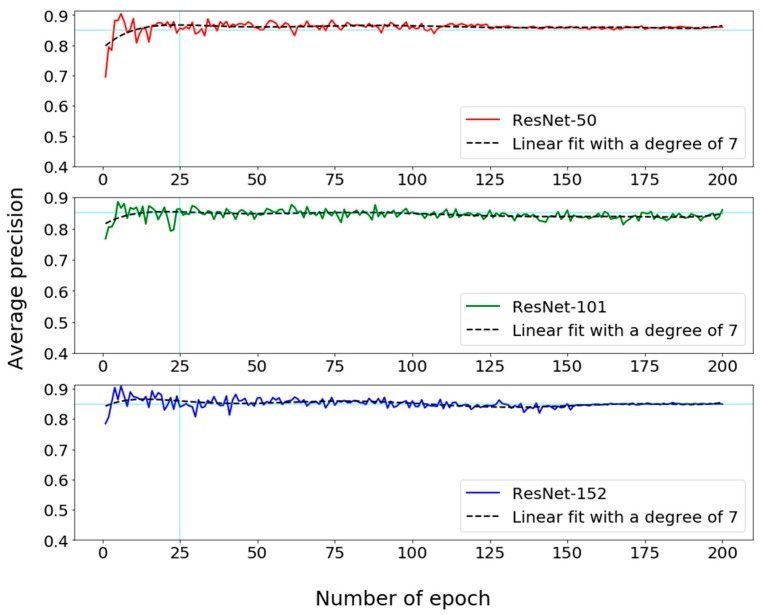
Validating the RetinaNet object detector learning with 50-layer, 101-layer, and 152-layer ResNets, respectively, for optimizing the RetinaNet object detector.

For testing the performance of using deep learning to estimate the locations of UPCs in GSV images, we conducted experiments on a customized server, which is equipped with an Intel i5 CPU, 16 GB RAM, a GeForce GTX 970 graphic card, and a GeForce GTX 1080ti graphic card. To obtain an optimal parameter setting, we trained and validated the RetinaNet object detector with 50-layer, 101-layer, and 152-layer ResNets (denoted as RetinaNet-50, RetinaNet-101, and RetinaNet-152, respectively) using the same labeled samples. For each of the three trained detectors (or detector-training choices), a step of 2500, a batch size of 1, and an epoch number of 200 were selected for the learning process. In the training step of DL, the step size was decided based on the sizes of batch and training dataset. During training, random horizontal flips augmentation along the X direction (with a chance of 0.5) was used to introduce variation in the training data. In order to avoid overfitting, a validation dataset was used to evaluate the accuracy of the RetinaNet object detector with three training choices at the end of each epoch. The strategies for reducing overfitting were suspended during the validation process. Figure 9 shows that the average precision (AP) reached the peak value around the 25th epoch and the model tended to be convergent. Thus, the RetinaNet object detectors were trained for 25 epochs before applying to utility pole detection from GSV images for our study area (see Figure 9).

### 4.2. Results and Discussions

#### 4.2.1. Accuracy Assessment of Object Detection

**Figure 10:**
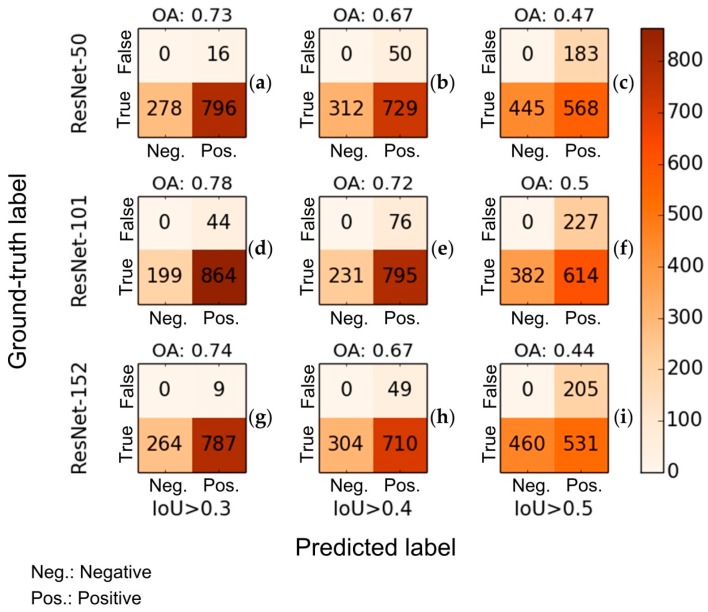
Confusion matrices for the RetinaNet object detector learning with 50-layer, 101-layer, and 152-layer ResNets after training 25 epochs on IoU with values greater than 0.3, 0.4, and 0.5 for choosing optimal parameters. (**a**) RetinaNet-50 with IoU > 0.3; (**b**) RetinaNet-50 with IoU > 0.4; (**c**) RetinaNet-50 with IoU > 0.5; (**d**) RetinaNet-101 with IoU > 0.3; (**e**) RetinaNet-101 with IoU > 0.4; (**f**) RetinaNet-101 with IoU > 0.5; (**g**) RetinaNet-152 with IoU > 0.3; (**h**) RetinaNet-152 with IoU > 0.4; (**i**) RetinaNet-152 with IoU > 0.5.

**Figure 11:**
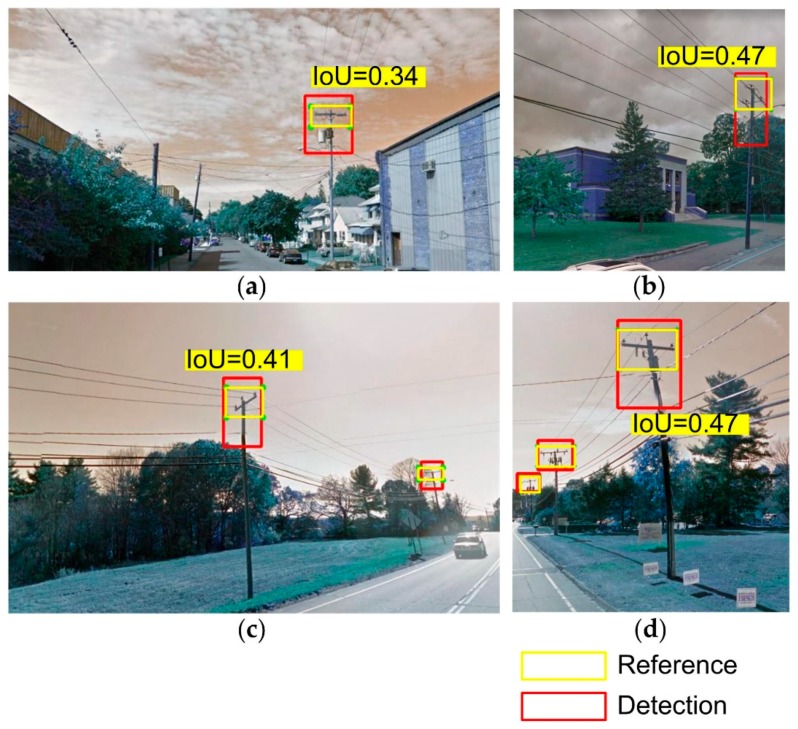
Examples of accuracy assessment of utility poles detection with underestimated performance at different measured IoUs. (**a**–**d**) are four different examples of true positive detections with different IoU values.

Figure 10 shows the accuracy of the RetinaNet object detector for detecting UPCs in GSV with 500 annotated GSV scenes including 937 reference objects. The count of false negative detection is zero in the confusion matrixes because non-utility pole objects were excluded from the training dataset. Overall, the RetinaNet-101 has the best overall accuracy (OA) among the three trained detectors. OAs of the RetinaNet-101 are around 78%, 72%, and 50% when the intersection-over-union (IoU) thresholds are greater than 0.3, 0.4, and 0.5, respectively. Additionally, compared with the other two trained detectors (RetinaNet-50 and RetinaNet-152), the RetinaNet-101 also has the highest recall and precision (Figure 10). The precisions of RetinaNet-101 are 0.95, 0.91, and 0.73 and its recalls are 0.81, 0.77, and 0.62, respectively, when IoUs thresholds are greater than 0.3, 0.4, and 0.5. It is worth mentioning that the OAs corresponding to different IoUs are presented because high IoUs may cause underestimation of the performance of the detection for UPCs in GSV in certain scenarios (Figure 11). For example, Figure 11 shows that the detector RetinaNet-101 was able to detect the target but still had an IoU less than the three selected IoU thresholds, which means the detected target was not considered as a positive detection. Based on the accuracy comparison, the RetinaNet-101 after training 25 epochs was finally chosen for detecting UPCs.

#### 4.2.2. Accuracy Assessment of Location Estimation

Table 1 shows the accuracy values of UPC location estimation, measured as the percentage of the number of estimated locations of UPCs located within the buffer zones of reference utility poles. To evaluate the impacts of the number of views, the threshold of angle, and the threshold of distance to the center of a selected road, we considered seven views (i.e., 3, 4, 5, 6, 7, 8, 9), three thresholds of angle (i.e., 1°, 2°, and 3°), and three thresholds of distance to the center of the selected road (i.e., 3 m, 4 m, and 5 m). For the method we tested, around half of the estimated UPC locations were within the 6 m buffer zone of their reference locations, and up to 79% of the estimated locations were within the 10 m buffer zone of reference locations. However, around 12% of the estimated UPC locations were within the 2 m buffer zone of reference locations which suggests that the LOB approach does not provide consistently accurate UPC locations. In terms of the threshold of angle in the modified brute-force-based three-station cross-location algorithm, Table 1 shows that using more views and larger angle thresholds resulted in more estimated UPCs, which is attributed to the increase of relaxation of the modified brute-force-based three-station cross location algorithm. In the meantime, more estimated UPCs could also result in lower accuracy in UPC estimation because relaxation allows more ghost nodes to be estimated UPCs (see Table 1). From Table 1, one can see that the average percentage of the number of estimated locations of UPCs being within all buffer zones of reference utility poles for the results of 8 views is the highest (47.80%) compared to the results of other numbers of views. In contrast, using larger thresholds of distance to center of selected road resulted in less estimated UPCs. In general, the accuracy of the location estimation for UPCs is reasonable and the estimated data are valuable because the optical GSV imagery was the only data source used to conduct the localization.

**Figure 12:**
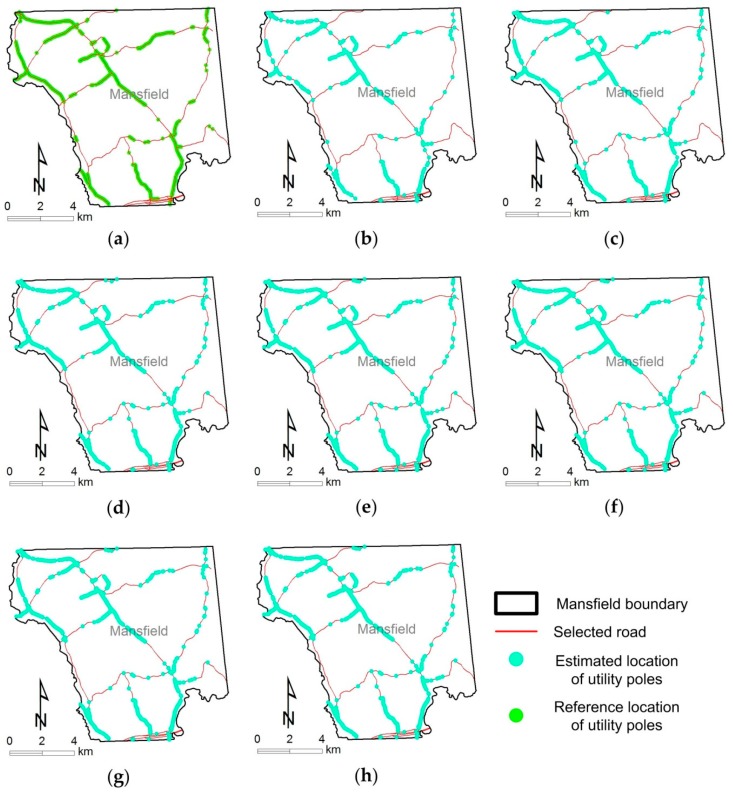
The distributions of reference utility poles with crossarms (UPCs) and estimated UPCs using different numbers of views, an angle threshold of 2°, and a distance threshold of 4 m in the study area. (**a**) Reference utility poles; (**b**) estimated UPCs using 3 views; (**c**) estimated UPCs using 4 views; (**d**) estimated UPCs using 5 views; (**e**) estimated UPCs using 6 views; (**f**) estimated UPCs using 7 views; (**g**) estimated UPCs using 8 views; (**h**) estimated UPCs using 9 views.

**Figure 13:**
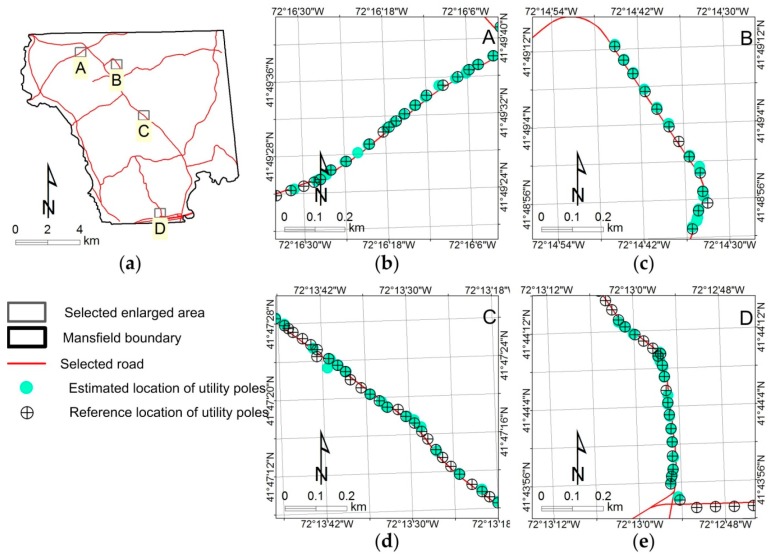
Enlarged examples of distribution patterns of reference and estimated utility poles with crossarms in the study area. (**a**) the locations of enlarged examples; (**b**) enlarged area A; (**c**) enlarged area B; (**d**) enlarged area C; (**e**) enlarged area D.

**Figure 14:**
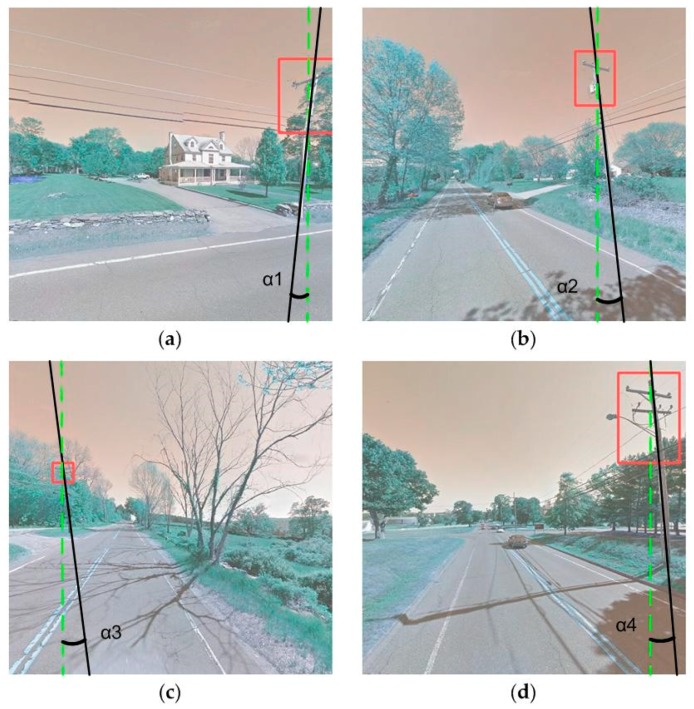
Examples of errors in estimating locations of utility poles from GSV, caused by the difference between ground locations (black solid lines) and their corresponding orthographic projected location (green dash lines). (**a**–**d**) are four different examples of leaning UPCs with different leaning angles.

Figure 12 shows a visual comparison of the distributions of estimated locations of UPCs with different numbers of views (i.e., 3, 4, 5, 6, 7, 8 and 9 views), an angle threshold of 2°, and a distance threshold of 4 m. Overall, the distributions of estimated UPC locations (Figure 12b–h) are almost the same as the distributions of the reference UPCs except for some missing UPCs (see Figure 13b–e). In particular, Figure 13b–e show that most estimated locations of UPCs are geographically close to the reference locations of UPCs. Our method failed to estimate UPC locations when a given UPC was not detected in at least three GSV images out of a certain number of views—three is the minimum number of images required to triangulate a position and eliminate ghost nodes (see Figure 7). This explains why the number of estimated UPCs increases as the number of views increases (see Table 1 and Figure 12b–h). Location mismatches that occurred (see Figure 13b–e) may have been caused by GSV image distortion, terrain relief, the position accuracy of GSV, UPC lean, or by limitations in the method we used. For example, ground locations of UPCs are different from the orthographic projected locations estimated from GSV images (see Figure 14) due to leaning UPCs. Our proposed method has great potential for the areas where GSV imagery is available and where a UPC distribution map with a ≤10 m accuracy is acceptable.

#### 4.2.3. Parameter Sensitivity Analysis of Location Estimation

To quantify the degree to which the location inference method affects the location estimation, we conduct Sobol’s sensitivity analysis with a sampling size of 500 on the three main parameters for estimating locations of UPCs (i.e., number of views, angle threshold, and buffer distance). Parameter sensitivity for the location estimation method (as a function) is analyzed through conducting experiments using a variety of combinations of parameters (Table 1). During the sampling process in Sobol’s sensitivity analysis, we simplified the testing method (i.e., location inference method) by grouping results into categories because of the time intensive execution of the testing method with hundreds of sampling scenarios. Two sets of sensitivity analysis were conducted from the aspect of parameters versus the number of estimated UPCs (Table 2) and parameters versus the percentage of the number of estimated locations of UPCs being within a 5 m buffer zone of reference UPCs (Table 3).

Table 2 exhibits that, after measuring sensitivity across the three parameters, the number of views contributes the most to the number of estimated UPCs, followed by angle threshold. More UPCs can be estimated as the number of views increases (Table 1). The threshold of distance shows very limited impact on the number of estimated UPCs. For example, in the case of the number of views being 8, the corresponding average numbers of estimated UPCs for angle thresholds 1°, 2°, and 3° are 636, 773, and 842, respectively; in contrast, the corresponding average numbers of estimated UPCs for distance thresholds 3 m, 4 m, and 5 m are 763, 763, and 726, respectively. However, Sobol’s sensitivity analysis on the percentage of the number of estimated UPC locations being within a 5 m buffer zone of reference UPCs shows that the effect of distance threshold is much stronger, increasing from 0.0157 to 0.1826 in terms of total order sensitivity (Table 3). This indicates that increasing the threshold of distance can reduce the error of location estimation by eliminating ghost nodes generated from almost parallel LOBs. Increasing the other two parameters has a similar effect because increasing the number of views and threshold of angle allows more LOB intersections to suffice conditions of being candidates.

#### 4.2.4. Limitations and Future Studies

This study is mainly an exploratory study, with only one method being used for each task of detection and location inference. Even though this study presents a great potential of using DL to map UPCs from GSV images, there are some limitations worth a mention. First, GSV is increasing the coverage and accelerating the update frequency, but it is still challenging to map up-to-date geographic information from GSV images. For example, the UPC locations may have changed since the time that the available GSV images were acquired, especially if the images are more than a few years old. Second, a large amount of training dataset is needed to achieve an acceptable accuracy with the Retina-101. Therefore, a comprehensive study about the minimum requirement of the training dataset for using DL to map UPCs or other geographic objects is needed. Third, we only validated the DL model on detecting utility poles with crossarms; however, a large percentage of utility poles do not have crossarms or other distinctive features. Fourth, accuracy of the LOB method is reduced when UPCs have a significant lean mainly because of terrain and optical distortion of GSV without compensation; better positions could have been obtained by detecting the base of the UPCs, but this would likely have resulted in false positives due to trees (Figure 14). Fifth, this method considers UPCs that are geographically very close to each other as one UPC because of the fundamental flaw of LOB measurement.

## 5. Conclusions

Mapping UPCs is a labor-intensive and time-consuming task. This study suggested a DL-based automatic mapping method to map UPCs from GSV, which uses a DL algorithm (the RetinaNet object detector) and the LOB measurement to estimate the locations of UPCs in GSV automatically. A case study was conducted to demonstrate the performance of the suggested method. The results show that (1) with properly-set parameters, the RetinaNet object detector is able to detect most UPCs from GSV (e.g., an OA of 0.78 can be achieved by RetinaNet-101 when IoU threshold is greater than 0.3); and (2) by combining LOB measurement and multiple points aggregation, the UPC position inference method can estimate the positions of UPCs at a reasonable accuracy (i.e., ≤10 m). In general, the suggested integrative method shows to be promising in our case study. With the wide availability of GSV images, the method might be a valuable way for automatically mapping UPCs and could be useful for mapping other geographic objects located along roads.

## Figures and Tables

**Table 1:** Accuracy assessment of location estimation of utility poles with crossarms (UPCs) based on 1039 reference UPCs.

Number of Views	Threshold of Angle (°)	Threshold of Distance to Center of Selected Road (m)	Percentage of the Number of Estimated Locations of UPCs Being within a Certain Buffer Zone of Reference Utility Poles (%)										Number of Estimated UPCs
			<1 m	<2 m	<3 m	<4 m	<5 m	<6 m	<7 m	<8 m	<9 m	<10 m	
**3**	1	3	1.75	8.04	22.38	35.66	46.85	54.9	62.59	68.18	74.83	80.07	286
	1	4	1.83	8.42	23.44	36.63	47.62	55.68	64.47	70.7	76.92	82.42	273
	1	5	1.92	7.69	23.08	37.31	49.23	57.69	67.31	72.69	79.23	85	260
	2	3	1.71	8.05	19.51	30.98	44.88	53.41	58.78	64.63	72.93	77.8	410
	2	4	1.75	8.27	19.8	31.08	45.36	53.88	60.15	66.92	74.94	79.95	399
	2	5	1.85	8.71	20.32	32.72	47.76	56.73	63.59	69.66	77.31	82.32	379
	3	3	2.37	8.84	20.47	31.9	43.32	50.86	57.54	64.01	71.12	75.86	464
	3	4	2.68	8.95	20.36	31.77	43.18	51.23	58.17	65.55	72.93	77.18	447
	3	5	2.56	9.3	20	32.79	44.88	53.02	60.23	66.98	74.65	79.53	430
**4**	1	3	2	7.56	19.56	30.89	40.67	51.33	58	63.11	71.11	76	450
	1	4	1.87	7.26	20.37	30.91	42.62	53.86	60.89	67.45	74.71	79.16	427
	1	5	2.02	7.83	20.96	32.32	44.44	55.81	63.38	70.45	77.78	83.33	396
	2	3	1.31	7.86	17.84	29.13	39.44	48.12	54.99	62.52	67.76	72.83	611
	2	4	1.2	7.72	18.01	29.5	41.68	51.29	58.32	65.87	71.7	76.33	583
	2	5	1.09	8.56	18.58	30.78	43.53	53.37	60.47	68.12	74.32	79.05	549
	3	3	1.35	7.77	16.89	29	38.42	46.79	55.31	62.78	68.76	74.14	669
	3	4	1.39	8.19	18.08	30.45	40.96	49.15	57.5	65.84	71.56	76.82	647
	3	5	1.47	9.61	19.06	30.94	42.51	50.98	59.45	68.4	74.43	78.99	614
**5**	1	3	2.2	10.8	22.34	35.35	45.6	51.83	57.51	64.29	71.98	77.47	546
	1	4	2.46	11	22.35	36.36	48.48	55.3	61.93	68.94	75.19	79.17	528
	1	5	2.63	11.3	22.63	37.37	49.7	56.97	63.84	71.92	77.78	83.23	495
	2	3	2.31	11	19.62	30.3	42.14	48.77	57.58	64.36	71.28	75.47	693
	2	4	2.53	10.3	19.2	32.44	45.83	53.27	61.61	68.3	73.66	77.98	672
	2	5	2.52	10.2	19.69	32.6	47.72	55.28	63.15	70.08	77.17	81.57	635
	3	3	2.37	10.4	19.05	29.17	39.55	47.83	56.37	63.34	69.51	74.24	761
	3	4	2.84	10.4	19.08	30.72	42.63	51.42	60.35	66.98	72.26	76.73	739
	3	5	3.01	11.1	20.23	32.28	43.9	53.52	62.41	70.01	76.33	80.63	697
**6**	1	3	2.5	12.2	23.21	36.06	46.41	52.92	60.27	67.78	73.12	78.46	599
	1	4	2.87	12.2	23.99	37.67	48.14	54.56	63.18	70.78	74.32	78.55	592
	1	5	2.5	12.9	25.04	38.64	49.91	57.07	65.47	73.7	78	82.47	559
	2	3	2.43	10.8	21.62	33.92	44.73	52.3	59.86	65.14	71.76	78.24	740
	2	4	2.46	10.3	21.61	34.75	47.74	56.5	64.71	70.86	74.69	79.07	731
	2	5	2.47	11.2	23.11	36.63	50.44	59.88	67.3	73.4	78.05	82.27	688
	3	3	2.22	9.75	20.49	32.47	41.23	50.49	57.9	63.21	70.62	75.43	810
	3	4	2.63	10.1	21.13	33.88	44.5	55.38	62.88	68.63	73.25	76.63	800
	3	5	2.76	10.8	22.97	34.78	46.19	57.09	64.44	70.47	76.12	79.4	762
**7**	1	3	2.7	12.4	24.01	38.31	49.92	56.6	63.28	68.68	74.72	79.81	629
	1	4	2.91	12.4	25.36	41.03	52.67	58.16	65.59	72.54	77.71	82.23	619
	1	5	2.74	13.2	27.05	43.15	55.65	60.96	69.01	75.34	80.65	84.93	584
	2	3	2.2	9.95	21.71	34.24	44.44	52.07	59.56	65.37	70.8	76.36	774
	2	4	2.61	9.52	23.21	36.64	47.72	56.19	64.15	70.01	73.14	77.84	767
	2	5	2.37	10.6	23.29	38.35	51.05	59.14	67.78	73.08	77.82	82.01	717
	3	3	2.75	10.2	22.04	33.53	43.95	52.22	59.28	66.35	72.1	75.57	835
	3	4	3.15	10.2	23.12	35.23	46.25	55.33	62.47	68.77	72.88	76.15	826
	3	5	3.31	11.5	23.92	36.01	48.6	56.87	65.14	70.99	75.95	79.64	786
**8**	1	3	3.08	12.2	25.08	40.92	52.92	58.77	64.77	70.46	70.46	76.77	650
	1	4	3.87	12.9	26.63	43.03	55.42	60.84	66.41	73.68	78.17	82.51	646
	1	5	4.08	13.7	28.22	45.02	58.4	63.62	70.47	77.16	82.54	86.79	613
	2	3	3.44	11.3	23.66	36.01	47.2	54.33	61.58	65.52	70.74	75.7	786
	2	4	3.68	11.3	25.51	37.44	50	57.61	64.47	69.16	72.21	76.78	788
	2	5	3.49	12.1	26.71	39.19	52.62	59.46	67.65	72.89	77.18	80.54	745
	3	3	3.05	11.2	23.12	34.62	45.42	52.93	60.09	66.2	71.24	75.7	852
	3	4	3.51	11.2	24.09	35.79	47.6	56.02	61.99	69.36	72.4	76.02	855
	3	5	3.78	13.3	25.61	38.66	49.88	57.44	65.73	71.71	76.1	79.63	820
**9**	1	3	2.67	11.7	24.67	41.46	52.15	58.99	65.53	71.92	76.37	82.91	673
	1	4	2.85	12	26.39	42.73	54.72	61.62	66.87	73.01	77.21	82.01	667
	1	5	3.14	13.8	28.57	45.84	58.87	64.52	70.8	76.77	81.16	85.71	637
	2	3	3.18	11.3	22.03	36.72	47.37	54.59	61.57	65.97	70.26	75.64	817
	2	4	3.04	12.2	23.45	37.3	48.97	57.23	62.33	67.8	71.08	75.7	823
	2	5	3.47	12.5	25.06	40.36	51.8	59.13	66.07	71.47	75.45	78.92	778
	3	3	2.95	11.4	22.05	36.36	47.16	54.77	60.91	65.8	70.11	75.34	880
	3	4	2.83	12.2	23.42	36.88	49.21	56.56	62.44	67.99	71.15	75.23	884
	3	5	3.4	12.9	24.74	39.98	51.11	58.15	64.95	71.04	75.38	78.55	853

**Table 2:** Sobol’s sensitivity analysis on the number of estimated UPCs based on tested results

Sobol (N = 500)				
Parameter	S1	S1_conf	ST	ST_conf
Number of views	0.6786	0.0979	0.6851	0.0730
Threshold of angle	0.2921	0.060	0.2962	0.0299
Threshold of distance	0.0150	0.0160	0.0157	0.0021

S1: First order sensitivity; ST: Total order sensitivity; conf: corresponding confidence intervals with a confidence level of 95%.

**Table 3:** Sobol’s sensitivity analysis on the percentage of the number of estimated locations of UPCs being within a 5 m buffer zone of reference UPCs based on tested results.

Sobol (N = 500)				
Parameter	S1	S1_conf	ST	ST_conf
Number of views	0.5084	0.08334	0.6066	0.0779
Threshold of angle	0.2185	0.05645	0.2841	0.03889
Threshold of distance	0.15367	0.0500	0.1826	0.0274

S1: First order sensitivity; ST: Total order sensitivity; conf: corresponding confidence intervals with a confidence level of 95%.
